# Predicting Immunotherapy-Induced Pneumonitis Based on Chest CT and Non-Imaging Data

**DOI:** 10.3390/cancers17182980

**Published:** 2025-09-12

**Authors:** Qing Lyu, Hongyu Yuan, Zhen Lin, Janardhana Ponnatapura, Christopher T. Whitlow

**Affiliations:** 1 Department of Radiology, Wake Forest University School of Medicine, Winston-Salem, NC 27103, USA; hongyu.yuan@advocatehealth.org (H.Y.); christopher.whitlow@advocatehealth.org (C.T.W.); 2 Department of Biomedical Engineering, Wake Forest University School of Medicine, Winston-Salem, NC 27103, USA; zhen.lin@wfusm.edu

**Keywords:** radiomics, deep learning, medical image analysis, lung segmentation, immune checkpoint inhibitors, non-small cell lung cancer

## Abstract

**Background/Objectives:** Immune checkpoint inhibitors (ICIs) have been extensively used for the treatment of non-small cell lung cancer patients in recent years, providing a significant survival benefit. However, a major drawback of ICI-related immunotherapy is the risk of developing post-surgical pneumonitis. **Methods**: In this study, we propose a deep learning-embedded, multi-modality prediction approach to assess whether patients will develop ICI-pneumonitis after receiving ICI-based immunotherapy. This approach utilizes multi-modal data, including clinical data and pre-treatment lung screening computed tomography (CT) images. We extracted three types of features: (1) deep learning features from CT scans using a pre-trained vision transformer; (2) radiomic features from CT scans using pre-defined radiomic algorithms; (3) clinical features from patients’ electronic health records. We then compared ten machine learning algorithms for prediction based on these extracted features. **Results**: Our experiments demonstrated that using all three types of features leads to the best prediction result, with a prediction accuracy rate of 0.823 and an area under the receiver operating characteristic curve of 0.895. **Conclusion:** Multimodal approaches can result in superior prediction results compared to single modality approaches. This study demonstrates the feasibility of developing machine learning algorithms to accurately predict ICI-pneumonitis and contributes to the early identification of patients who are at a higher risk of developing pneumonitis.

## Simple Summary

Immune checkpoint inhibitors improve survival in non-small cell lung cancer but carry a risk of post-surgical pneumonitis. This study presents a deep learning-embedded, multi-modality prediction approach to identify patients at risk of immune checkpoint inhibitors-related pneumonitis. The approach integrates three feature types: (1) deep learning features from pre-treatment CT scans using a pretrained vision transformer; (2) radiomic features extracted via predefined algorithms; (3) clinical data from patient records. Ten machine learning algorithms were evaluated, with the combined multimodal feature set achieving the highest performance. Results indicate that combining imaging-derived and clinical features yields superior predictions compared to single-modality models. This work demonstrates the feasibility of leveraging machine learning to enhance early detection of pneumonitis risk in non-small cell lung cancer patients receiving immune checkpoint inhibitors, potentially enabling timely clinical interventions and improved patient outcomes.

## 1. Introduction

Lung cancer is the most frequently diagnosed cancer and the leading cause of cancer death worldwide [1]. Non-small cell lung cancer (NSCLC) is the most common subtype, accounting for approximately 85% of all lung cancer cases [2]. Recently, immunotherapy, particularly using immune checkpoint inhibitors (ICIs), has demonstrated superior outcomes in the treatment of NSCLC, significantly improving overall survival compared to chemotherapy [3,4]. ICIs provoke immune reactions against cancer cells by blocking inhibitory receptors such as programmed cell death protein-1 (PD-1), programmed death-ligand 1 (PD-L1), and cytotoxic T-lymphocyte antigen 4 (CTLA-4) [5,6,7]. However, ICIs can also induce autoimmune reactions that are harmful to healthy tissues by disrupting normal immune system homeostasis, resulting in immune-related adverse events (irAEs) [8]. ICI-related pneumonitis (ICI-P) is a rare but life-threatening irAE, with an overall incidence range of 3–6% and a mortality range of 22–33% for severe cases (grade 3–4) [9,10,11]. ICI-P can potentially cause significant morbidity, leading to the discontinuation of therapy and even mortality [8]. Clinical diagnosis of ICI-P is challenging due to its nonspecific symptoms and similarities to other pulmonary conditions [12]. There is no gold standard for clinical diagnosis, making it necessary to develop pretreatment ICI-P prediction methods.

Multiple studies have investigated the feasibility of predicting ICI-P before NSCLC patients receive immunotherapy. Risk factors such as the tumor histology type, ICI selection, combination therapy, preexisting diseases (e.g., interstitial lung disease and extrathoracic metastasis), smoking history, and radiotherapy history are believed to influence the incidence rate of ICI-P [10,13,14,15,16,17,18,19]. Based on these findings, several prediction models have been developed. Jia et al. proposed a dynamic online hypertension nomogram to predict ICI-P for NSCLC patients [20]. Gong et al. developed a machine learning algorithm to identify and predict ICI-P based on eleven predictors [21]. Li and Xu both built risk assessment nomograms for ICI-P prediction [22,23]. In addition to using risk factors, researchers hypothesize that chest CT images contain discriminative information for ICI-P prediction. To validate this hypothesis, Colen et al. proposed a radiomics-based ICI-P prediction method, extracting 1860 radiomic features from chest computed tomography (CT) images [24]. Mu et al. developed a radiomics nomogram to predict severe irAEs using fluorine-18 fluorodeoxyglucose positron emission tomography (PET) and CT images [25]. Cheng et al. created a deep learning-embedded nomogram approach for ICI-P prediction, where a CT score, calculated from five radiology features extracted by a neural network, was input into a nomogram along with three other features for the final prediction [26]. Tan et al. constructed a multimodal deep learning model based on 3D CT images and clinical data, achieving an overall accuracy rate of 0.92 for ICI-P prediction through five-fold cross-validation [27]. Additionally, chest CT images can help discriminate different types of pneumonitis. Tohidinezhad et al. explored the feasibility of establishing a prediction model to differentiate ICI-P from other types of pneumonitis in NSCLC patients undergoing immunotherapy [28]. Mallio et al. utilized a deep learning algorithm based on chest CT images to distinguish coronavirus disease 2019 (COVID-19) pneumonia from ICI-P [29].

Differing from existing studies, we propose a multimodal ICI-P prediction model utilizing three types of features: deep learning features, radiomic features, and clinical features. Deep learning features are extracted from a pretrained vision transformer (ViT), radiomic features are derived from predefined radiomic algorithms, and clinical features are obtained from a clinical patient database. After feature selection, the selected features are input into the proposed ICI-P prediction model to predict the likelihood of ICI-P development following immunotherapy.

## 2. Materials and Methods

### 2.1. Dataset

We collected data from 1254 NSCLC patients who received immunotherapy between 2005 and 2021 at Wake Forest Baptist Medical Center. Among these, 51 patients developed ICI-P and were included in the experimental group for this study. Conversely, we randomly selected 41 patients who did not have ICI-P after immunotherapy to serve as the control group. Table 1 presents the demographic information of the patients. For each patient, we collected both the most recent chest CT scan taken before the first immunotherapy session and the corresponding clinical data. This study was approved by the Wake Forest University School of Medicine Institutional Review Board (IRB), and all patient data used in this research were collected and analyzed in compliance with the ethical standards set by the board.

### 2.2. Image Processing

**Figure 1:**
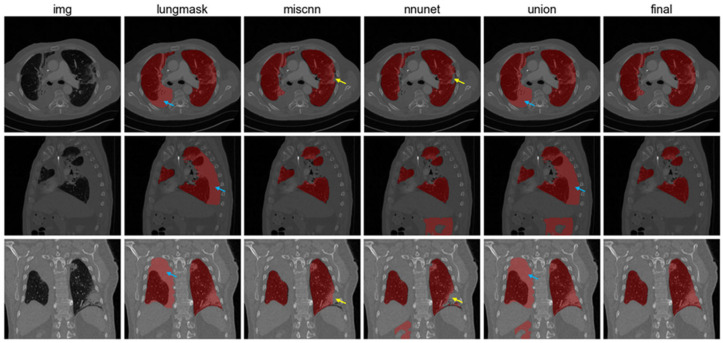
Lung segmentation results. From left to right: CT images, Lungmask segmentation, COVID-19 MIScnn, nnUNet, union results, and final results after manual correction. Blue arrows show false positive regions and yellow arrows demonstrate false negative regions.

We first preprocessed the CT images to unify the voxel resolution to 1 × 1 × 1 mm using nearest interpolation and cropped each image to a size of 512 × 512. At the same time, the pixel intensity was truncated to a range between -2100 and 100 Hounsfield units (HU). Lung segmentation was then implemented using three different networks: Lungmask [30], nnUNet [31], and COVID-19 MIScnn [32]. To achieve accurate lung segmentation, we developed a strategy that combines the results from these three networks. First, we created a union of the results from Lungmask, nnUNet, and COVID-19 MIScnn. Then, we manually checked each result from the previous step and corrected misclassified regions. The results were assessed by three experienced radiologists to mitigate subjective bias (with 6, 8, and 13 years of experience, respectively). Figure 1 demonstrates the comparison of our segmentation method with other methods, including three individual models (Lungmask, nnUnet, and MIScnn) and the union of three individual models.

### 2.3. Vision Transformer

Deep learning is a data-driven approach in which neural networks automatically learn high-level representations directly from image pixel data. These multilayer networks transform input pixels into increasingly abstract features optimized for the target task during training, rather than relying on predefined expert-designed descriptors. Given the huge success of transformers in natural language processing, ViT have recently been proposed as a competitive alternative to convolutional neural networks for various computer vision tasks, such as image classification, object detection, and semantic image segmentation [33]. In this study, we pretrained a ViT model to extract deep learning features from 3D CT images. A ViT-base model was pretrained with a hidden size of 768, 12 heads, and a depth of 12, following the strategy outlined by Niu et al. [34].

During deep learning feature extraction, the CT images were divided into multiple 4 × 16 × 16 patches and fed into the pretrained ViT model. Each input patch produced a vector with a dimension of 768, and the summation of all vectors was used as the final set of deep learning features. For each patient, there were 768 deep learning features extracted.

### 2.4. Radiomics

Differing from deep learning, radiomic features are handcrafted, predefined quantitative descriptors extracted from segmented regions of interest. Through high-throughput extraction, radiomics quantifies the tissue heterogeneity, shape, intensity distributions, and texture patterns that extend beyond human visual perception. We utilized the Python Pyradiomics library for radiomic feature extraction [35]. To enhance the utilization of information within CT images, a wavelet transform was applied before the feature extraction process. For each patient, there were 863 radiomic features extracted, categorized into 8 major categories: first order statistics, 2D shape-based, 3D shape-based, gray-level co-occurrence matrix, gray-level run length matrix, gray-level size zone matrix, neighboring gray tone difference matrix, and gray-level dependence matrix categories.

### 2.5. Non-Imaging Data Preprocessing

Following our non-imaging feature selection strategy—(1) excluding features with incomplete information and (2) excluding features not recorded in both the experimental and control groups—ten clinical features were extracted from patients’ electronic health record (EHR) data: (1) pack years; (2) age; (3) body mass index (BMI); (4) baseline oxygen dependence; (5) whether the patient had received surgery prior to immunotherapy; (6) whether the patient had received radiation prior to immunotherapy; (7) Eastern Cooperative Oncology Group Performance Status (ECOG-PS) at the time of immunotherapy; (8) choice of immunotherapy; (9) whether immuno-oncology (IO) was given concurrently with chemotherapy; (10) total cycles of IO given. Since some clinical features are categorical and not represented numerically, we converted these features to one-hot encoding for prediction. For continuous features, we simply normalized them into the range of zero to one.

### 2.6. Feature Selection

After feature extraction, we obtained a total of 768 deep learning features, 863 radiomic features, and 10 clinical features. To remove redundant and non-relevant features, we performed feature selection using the Chi-square test and Student’s *t*-test. For deep learning and radiomic features, we retained only the 25 most significant features of each type. All 10 clinical features were kept. As a result, a total of 60 features were utilized for prediction after feature selection.

### 2.7. Machine Learning Prediction Model

We utilized the Python Pycaret library to establish ICI-P models. Ten different machine learning prediction models were compared, including logistic regression, K-neighbors classifier, support vector machine, gradient boosting classifier, AdaBoost classifier, decision tree classifier, light gradient boosting machine, extra trees classifier, naïve bayes, and random forest classifier models.

### 2.8. Experimental Details

We pretrained the ViT model on 2000 chest CT scans collected by Wake Forest Baptist Medical Center between 2015 and 2021. The pretraining process was carried out for 100 epochs on an Nvidia A100 GPU(Nvidia Corporation, Santa Clara, CA, USA). During pretraining, the original CT images were divided into multiple smaller patches measuring 64 by 64 by 64. When combining features extracted from different approaches, we first normalized each feature to standardize feature values, mitigating potential issues caused by mismatched feature value magnitudes. Clinical features were processed through either one-hot encoding or manual labeling to convert discrete features into a continuous domain. When using PyCaret for ICI-P prediction, three-fold cross validation was employed.

## 3. Results

### 3.1. Patient Characteristics

We pretrained the ViT model on 2000 chest CT scans collected by Wake Forest Baptist Medical Center between 2015 and 2021. The pretraining process was carried out for 100 epochs on an Nvidia A100 GPU. During pretraining, the original CT images were divided into multiple smaller patches of size 64 by 64 by 64. When combining features extracted from different approaches, we first normalized each feature to standardize feature values, mitigating potential issues caused by mismatched feature value magnitudes. Clinical features were processed through either one-hot encoding or manual labeling to convert discrete features into a continuous domain. When using PyCaret for ICI-P prediction, three-fold cross validation was employed.

A total of 92 NLCSC patients who received at least one cycle of immunotherapy at Wake Forest Baptist Medical Center were enrolled in this study. Among these patients, 51 developed ICI-P, with a median time of 149 days between the initial immunotherapy date and ICI-P diagnosis date. Of the patients who developed ICI-P, 45% received pembrolizumab, 31% received nivolumab, 14% received durvalumab, 4% received atezolizumab, and the remaining patients received a combination of immune checkpoint inhibitors. All ICI-P patients were diagnosed with pneumonitis of grade 2 or higher; 44% had grade 2, 44% had grade 3, 7% had grade 4, and 9% had grade 5 pneumonitis. The 41 patients who did not develop ICI-P were used as the control group. Among them, 62% received pembrolizumab, 17% received nivolumab, 5% received durvalumab, 14% received atezolizumab, and 2% received lenvatinib.

**Figure 2:**
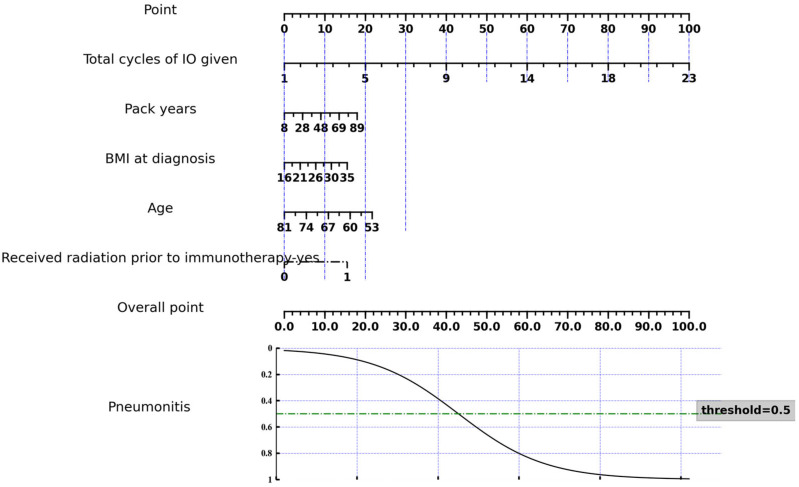
Nomogram of the ICI-P prediction model based on selected clinical features.

When conducting the Chi-square test and Student’s *t*-test for clinical feature selection, we identified four features, including total cycles of IO given, pack years, BMI at diagnosis, and age, which showed significant differences between ICI-P and control groups, as shown in Table 2. A nomogram was constructed as a quantitative method to predict the risk of ICI-P in NSCLC patients, as shown in Figure 2.

### 3.2. ICI-Pneumonitis Prediction Results Based on All Types of Features

Metrics such as the accuracy, area under the receiver operating characteristic curve (AUC), recall, precision, and F1 score were utilized to evaluate the performance of the ICI-P prediction models. Table 3 presents a comparison of different prediction models generated using PyCaret based on all selected multi-modal features. It can be seen that logistic regression achieved the best performance in terms of accuracy, AUC, recall, and F1 score. Meanwhile, support vector machines achieved the highest precision.

### 3.3. Feature Importance Analysis

**Figure 3:**
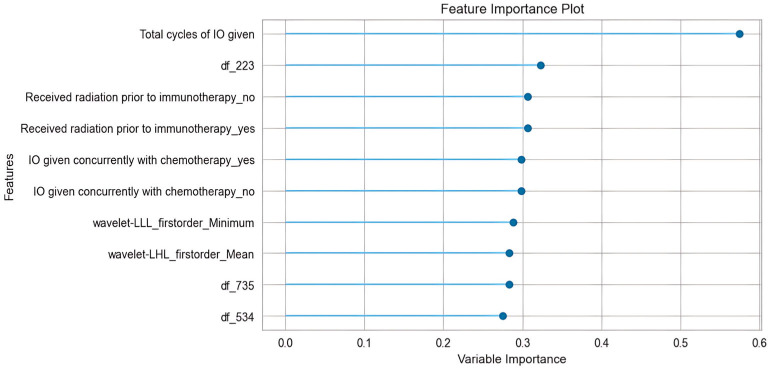
Feature importance plot.

We conducted a feature importance analysis and explored those features that contributed most to the prediction results. Figure 3 shows a feature importance plot of the most important features. Among the top ten most contributive features, there are five clinical, three deep-learning, and two radiomic features. It can be seen that the most influential predictor is the feature “total cycles of IO given,” with a much larger importance value than the rest, indicating the model’s performance degrades the most when this variable is permuted, so it is the primary driver of predictions. Other contributors cluster closely with moderate importances—such as categorical indicators created by one-hot encoding (for example, “received radiation prior to immunotherapy:_yes/no”; “IO given concurrently with chemotherapy:_yes/no”) and several radiomic features (e.g., wavelet-LLL_firstorder_Minimum, df_223)—suggesting these add incremental signals beyond the dominant feature but are partially redundant with each other.

### 3.4. Ablation Study

We conducted an ablation study to assess the contribution of each modality of features to the final prediction results and to demonstrate the effectiveness of the proposed method. A series of experiments were performed, building prediction models under four situations: using clinical features alone, deep learning features alone, radiomic features alone, and a combination of all features. As shown in Table 4. The results indicate that using all three kinds of features together leads to higher accuracy and AUC scores compared to using each type of feature individually. The only exception was the random forest model, which performed better when the prediction was based solely on clinical features.

## 4. Discussion

Immune checkpoint inhibitor immunotherapy is a revolutionary treatment for NSCLC that leverages the body’s immune system to target and destroy cancer cells. ICIs significantly improve outcomes for NSCLC patients, leading to longer overall survival and durable responses for some patients compared to traditional chemotherapy [3,4]. However, a major side effect of ICI-related immunotherapy is the potential development of irAEs, particularly ICI-P, which although rare can be life-threatening. In this paper, we propose a multi-modal approach to predict the occurrence of ICI-P in patients undergoing ICI immunotherapy. The approach incorporates three types of features: clinical features from patients’ electronic health records and radiomic and deep learning features extracted from CT scans. Our study demonstrates that using all three types of features together yields the best predictive performance.

In our ablation study, we found that clinical features contributed more to the final prediction results compared to radiomic and deep learning features. This is expected, as clinical features are directly related to the patients’ health conditions and their treatment processes. In contrast, radiomic and deep learning features, extracted from CT scans, provide only implicit information about a patient’s health status. Our experiments showed that the best prediction results were achieved when all three types of features were combined. This suggests that CT scans provide additional valuable information beyond what is available in the patients’ electronic health records, and this information can be effectively extracted using radiomic and deep learning algorithms.

Developing methods to predict ICI-P can lead to improved treatment outcomes. Accurate prediction of ICI-P allows for the early identification of patients who are at a higher risk of developing pneumonitis. This enables proactive monitoring, early intervention, and potentially modifying or discontinuing treatment to prevent severe outcomes. Predicting ICI-P risk can also help oncologists personalize treatment regimens. For patients at high risk, alternative therapies, dose adjustments, or combination strategies that lower the risk may be considered. In addition, predicting and managing ICI-P early can help prevent unplanned treatment interruptions, ensuring patients can continue their cancer therapy as planned and potentially improve overall outcomes.

One major limitation of this study is the relatively small sample size, which restricts the statistical power of the predictive models and may limit the generalizability of the findings to broader NSCLC populations receiving immunotherapy. The limited number of ICI-P cases also increases the risk of overfitting, particularly when integrating high-dimensional imaging features from CT scans with diverse clinical variables. Additionally, the retrospective nature of the data collection may introduce selection bias and missing information, while variations in imaging protocols, follow-up intervals, and pneumonitis grading criteria across patients further complicate model standardization and validation processes. These constraints highlight the need for larger, multi-institutional datasets and prospective validation to strengthen the robustness and clinical applicability of the proposed algorithms. In the future, we will initiate a multi-institutional study to collect more ICI-P subjects and develop advanced deep learning prediction algorithms such as multimodal graph convolutional networks to take factors such as patient similarities into account for improved prediction results.

From a translational standpoint, successful clinical integration will require not only external validation and prospective testing but also thoughtful embedding of the model into oncology workflows—ideally as a decision-support tool that complements, rather than replaces, radiologist and clinician judgment. Practical considerations, such as integration with existing radiology reporting systems, interpretability of predictions, and clear communication of risk estimates to treating clinicians, will be crucial for ensuring real-world adoption and clinical utility. In the future, we will collaborate with clinicians to explore the feasibility of deployment of the proposed method.

## 5. Conclusions

In this study, we focused on a major irAE of ICI-related immunotherapy and proposed a multi-modality approach to predict the occurrence of ICI-P in patients initiating ICI therapy. We extracted two types of imaging features and one type of non-imaging features. For imaging feature extraction, we first pre-trained a ViT model on a large-scale lung CT dataset and used it to obtain deep learning features. Additionally, radiomic features were computed using predefined hand-crafted algorithms. Ten clinical features were extracted from patients’ EHR data and used as non-imaging inputs. We evaluated ten machine learning prediction algorithms and conducted ablation studies to assess the contribution of each feature type. Our results show that combining all three types of features produced superior predictive performance compared to any single-modality approach. The best prediction model achieved an accuracy rate of 0.823 and an area under the receiver operating characteristic curve of 0.895. Our study indicates the feasibility of developing machine learning algorithms to accurately predict ICI-P and highlights their potential in enabling early identification of patients at higher risk for pneumonitis.

## Figures and Tables

**Table 1:** Patient demographic information.

	ICI-P	Control
Amount	51	41
Gender	F: 21, M: 30	F: 26, M: 15
Age	62.3 ± 10.9	68.7 ± 11.4
BMI	28.4 ± 7.0	23.9 ± 5.3

**Table 2:** Identifying significant clinical features based on the Chi-square test and Student’s *t*-test.

Clinical Features		*p*-Value
Total cycles of IO given		<0.001
Pack years		<0.001
BMI at diagnosis		<0.001
Age		<0.001
Received radiation prior to immunotherapy	Yes	0.035
	No	0.072
ECOG PS at the time of immunotherapy		0.054
Choice of immunotherapy	Pembrolizumab	0.178
	Nivolumab	0.353
	Durvalumab	0.078
	Atezolizumab	0.453
	Other	0.381
Received surgery prior to immunotherapy	Yes	0.099
	No	0.402
Baseline oxygen dependence	Yes	0.130
	No	0.769
IO given concurrently with chemotherapy	Yes	0.141
	No	0.220

**Table 3:** Comparison of different ICI-pneumonitis prediction models.

Model	Accuracy	AUC	Recall	Precision	F1
Logistic regression	0.823	0.895	0.853	0.853	0.852
K-neighbors classifier	0.809	0.892	0.787	0.882	0.830
Support vector machines	0.783	0.824	0.664	0.975	0.775
Gradient boosting classifier	0.746	0.818	0.789	0.791	0.788
AdaBoost classifier	0.744	0.845	0.764	0.816	0.783
Decision tree classifier	0.709	0.722	0.662	0.811	0.725
Light gradient boosting machine	0.683	0.779	0.769	0.716	0.732
Extra trees classifier	0.683	0.711	0.811	0.714	0.757
Naïve Bayes	0.658	0.618	0.791	0.700	0.736
Random forest	0.631	0.726	0.767	0.672	0.714

**Table 4:** Comparison of multiple prediction models on different types of features.

Model	Accuracy				AUC			
	Clinical	Radiomic	Deep	All	Clinical	Radiomic	Deep	All
LR	0.802	0.721	0.711	0.823	0.835	0.723	0.757	0.895
KNN	0.790	0.691	0.655	0.809	0.826	0.712	0.671	0.892
SVM	0.723	0.677	0.688	0.783	0.794	0.692	0.692	0.824
GBC	0.727	0.661	0.644	0.746	0.792	0.712	0.677	0.818
RF	0.713	0.555	0.577	0.631	0.760	0.679	0.685	0.726

LR: linear regression, KNN: K-neighbors classifier, SVM: support vector machines, GBC: gradient boosting classifier, RF: random forest.

## Data Availability

The data presented in this study are available on request from the corresponding author. The data are not publicly available because they contain information that could compromise the privacy of patients.

## Abbreviations

The following abbreviations are used in this manuscript:

ICI	Immune checkpoint inhibitor
CT	Computed tomography
NSCLC	Non-small cell lung cancer
irAE	Immune-related adverse events
EHR	Electronic health record
ViT	Vision transformer
AUC	Area under the receiver operating characteristic curve

## References

[1] Bray F., Laversanne M., Sung H., Ferlay J., Siegel R.L., Soerjomataram I., Jemal A. (2024). Global cancer statistics 2022: GLOBOCAN estimates of incidence and mortality worldwide for 36 cancers in 185 countries. CA Cancer J. Clin..

[2] Molina J.R., Yang P., Cassivi S.D., Schild S.E., Adjei A.A. (2008). Non-small cell lung cancer: Epidemiology, risk factors, treatment, and survivorship. Mayo. Clin. Proc..

[3] Doroshow D.B., Sanmamed M.F., Hastings K., Politi K., Rimm D.L., Chen L., Melero I., Schalper K.A., Herbst R.S. (2019). Immunotherapy in Non–Small Cell Lung Cancer: Facts and Hopes. Clin. Cancer Res..

[4] Malhotra J., Jabbour S.K., Aisner J. (2007). Current state of immunotherapy for non-small cell lung cancer. Transl. Lung Cancer Res..

[5] Herbst R.S., Morgensztern D., Boshoff C. (2018). The biology and management of non-small cell lung cancer. Nature.

[6] D’Incecco A., Andreozzi M., Ludovini V., Rossi E., Capodanno A., Landi L., Tibaldi C., Minuti G., Salvini J., Coppi E. (2015). PD-1 and PD-L1 expression in molecularly selected non-small-cell lung cancer patients. Br. J. Cancer.

[7] Reck M., Rodríguez-Abreu D., Robinson A.G., Hui R., Csőszi T., Fülöp A., Gottfried M., Peled N., Tafreshi A., Cuffe S. (2016). Pembrolizumab versus Chemotherapy for PD-L1–Positive Non–Small-Cell Lung Cancer. N. Engl. J. Med..

[8] Kalisz K.R., Ramaiya N.H., Laukamp K.R., Gupta A. (2019). Immune Checkpoint Inhibitor Therapy–related Pneumonitis: Patterns and Management. RadioGraphics.

[9] Naidoo J., Wang X., Woo K.M., Iyriboz T., Halpenny D., Cunningham J., Chaft J.E., Segal N.H., Callahan M.K., Lesokhin A.M. (2017). Pneumonitis in Patients Treated With Anti–Programmed Death-1/Programmed Death Ligand 1 Therapy. J. Clin. Oncol..

[10] Wang D.Y., Salem J.E., Cohen J.V., Chandra S., Menzer C., Ye F., Zhao S., Das S., Beckermann K.E., Ha L. (2018). Fatal Toxic Effects Associated with Immune Checkpoint Inhibitors: A Systematic Review and Meta-analysis. JAMA Oncol..

[11] Tone M., Izumo T., Awano N., Kuse N., Inomata M., Jo T., Yoshimura H., Minami J., Takada K., Miyamoto S. (2019). High mortality and poor treatment efficacy of immune checkpoint inhibitors in patients with severe grade checkpoint inhibitor pneumonitis in non-small cell lung cancer. Thorac. Cancer.

[12] Gomatou G., Tzilas V., Kotteas E., Syrigos K., Bouros D. (2020). Immune Checkpoint Inhibitor-Related Pneumonitis. Respiration.

[13] Suresh K., Voong K.R., Shankar B., Forde P.M., Ettinger D.S., Marrone K.A., Kelly R.J., Hann C.L., Levy B., Feliciano J.L. (2018). Pneumonitis in Non–Small Cell Lung Cancer Patients Receiving Immune Checkpoint Immunotherapy: Incidence and Risk Factors. J. Thorac. Oncol..

[14] Lisberg A., Cummings A., Goldman J., Bornazyan K., Reese N., Wang T., Coluzzi P., Ledezma B., Mendenhall M., Hunt J. (2018). A Phase II Study of Pembrolizumab in EGFR-Mutant, PD-L1+, Tyrosine Kinase Inhibitor Naïve Patients With Advanced NSCLC. J. Thorac. Oncol..

[15] Shibaki R., Murakami S., Matsumoto Y., Yoshida T., Goto Y., Kanda S., Horinouchi H., Fujiwara Y., Yamamoto N., Kusumoto M. (2019). Association of immune-related pneumonitis with the presence of preexisting interstitial lung disease in patients with non-small lung cancer receiving anti-programmed cell death 1 antibody. Cancer Immunol. Immunother..

[16] Su Q., Zhu E.C., Wu J.-B., Li T., Hou Y.-L., Wang D.-Y., Gao Z.-H. (2019). Risk of Pneumonitis and Pneumonia Associated With Immune Checkpoint Inhibitors for Solid Tumors: A Systematic Review and Meta-Analysis. Front. Immunol..

[17] Cho J.Y., Kim J., Lee J.S., Kim Y.J., Kim S.H., Lee Y.J., Cho Y.-J., Yoon H.I., Lee J.H., Lee C.-T. (2018). Characteristics, incidence, and risk factors of immune checkpoint inhibitor-related pneumonitis in patients with non-small cell lung cancer. Lung Cancer.

[18] Zhai X., Zhang J., Tian Y., Li J., Jing W., Guo H., Zhu H. (2020). The mechanism and risk factors for immune checkpoint inhibitor pneumonitis in non-small cell lung cancer patients. Cancer Biol. Med..

[19] Lin M.-X., Zang D., Liu C.-G., Han X., Chen J. (2024). Immune checkpoint inhibitor-related pneumonitis: Research advances in prediction and management. Front. Immunol..

[20] Jia X., Chu X., Jiang L., Li Y., Zhang Y., Mao Z., Liang T., Du Y., Xu L., Shen Y. (2022). Predicting checkpoint inhibitors pneumonitis in non-small cell lung cancer using a dynamic online hypertension nomogram. Lung Cancer.

[21] Gong L., Gong J., Sun X., Yu L., Liao B., Chen X., Li Y.-S. (2023). Identification and prediction of immune checkpoint inhibitors-related pneumonitis by machine learning. Front. Immunol..

[22] Li X., Lv F., Wang Y., Du Z. (2022). Establishment and validation of nomogram for predicting immuno checkpoint inhibitor related pneumonia. BMC Pulm. Med..

[23] Xu H., Feng H., Zhang W., Wei F., Zhou L., Liu L., Zhao Y., Lv Y., Shi X., Zhang J. (2022). Prediction of immune-related adverse events in non-small cell lung cancer patients treated with immune checkpoint inhibitors based on clinical and hematological markers: Real-world evidence. Exp. Cell Res..

[24] Colen R.R., Fujii T., Bilen M.A., Kotrotsou A., Abrol S., Hess K.R., Hajjar J., Suarez-Almazor M.E., Alshawa A., Hong D.S. (2017). Radiomics to predict immunotherapy-induced pneumonitis: Proof of concept. Investig. New Drugs.

[25] Mu W., Tunali I., Qi J., Schabath M.B., Gillies R.J. (2020). Radiomics of ^18^F Fluorodeoxyglucose PET/CT Images Predicts Severe Immune-related Adverse Events in Patients with NSCLC. Radiol. Artif. Intell..

[26] Cheng M., Lin R., Bai N., Zhang Y., Wang H., Guo M., Duan X., Zheng J., Qiu Z., Zhao Y. (2023). Deep learning for predicting the risk of immune checkpoint inhibitor-related pneumonitis in lung cancer. Clin. Radiol..

[27] Tan P., Huang W., Wang L., Deng G., Yuan Y., Qiu S., Ni D., Du S., Cheng J. (2022). Deep learning predicts immune checkpoint inhibitor-related pneumonitis from pretreatment computed tomography images. Front. Physiol..

[28] Tohidinezhad F., Bontempi D., Zhang Z., Dingemans A.-M., Aerts J., Bootsma G., Vansteenkiste J., Hashemi S., Smit E., Gietema H. (2023). Computed tomography-based radiomics for the differential diagnosis of pneumonitis in stage IV non-small cell lung cancer patients treated with immune checkpoint inhibitors. Eur. J. Cancer.

[29] Mallio C.A., Napolitano A., Castiello G., Giordano F.M., D’Alessio P., Iozzino M., Sun Y., Angeletti S., Russano M., Santini D. (2021). Deep Learning Algorithm Trained with COVID-19 Pneumonia Also Identifies Immune Checkpoint Inhibitor Therapy-Related Pneumonitis. Cancers.

[30] Hofmanninger J., Prayer F., Pan J., Röhrich S., Prosch H., Langs G. (2020). Automatic lung segmentation in routine imaging is primarily a data diversity problem, not a methodology problem. Eur. Radiol. Exp..

[31] Isensee F., Jaeger P.F., Kohl S.A.A., Petersen J., Maier-Hein K.H. (2020). nnU-Net: A self-configuring method for deep learning-based biomedical image segmentation. Nat. Methods.

[32] Müller D., Soto-Rey I., Kramer F. (2021). Robust chest CT image segmentation of COVID-19 lung infection based on limited data. Informatics Med. Unlocked.

[33] Han K., Wang Y., Chen H., Chen X., Guo J., Liu Z., Tang Y., Xiao A., Xu C., Xu Y. (2022). A Survey on Vision Transformer. IEEE Trans. Pattern Anal. Mach. Intell..

[34] Niu C., Lyu Q., Carothers C.D., Kaviani P., Tan J., Yan P., Kalra M.K., Whitlow C.T., Wang G. (2025). Medical multimodal multitask foundation model for lung cancer screening. Nat. Commun..

[35] Van Griethuysen J.J.M., Fedorov A., Parmar C., Hosny A., Aucoin N., Narayan V., Beets-Tan R.G.H., Fillion-Robin J.C., Pieper S., Aerts H.J.W.L. (2017). Computational Radiomics System to Decode the Radiographic Phenotype. Cancer Res..

